# Food and Waterborne Cryptosporidiosis from a One Health Perspective: A Comprehensive Review

**DOI:** 10.3390/ani14223287

**Published:** 2024-11-14

**Authors:** Munwar Ali, Yaru Ji, Chang Xu, Qazal Hina, Usama Javed, Kun Li

**Affiliations:** 1 Institute of Traditional Chinese Veterinary Medicine, College of Veterinary Medicine, Nanjing Agricultural University, Nanjing 210095, China; drmunwarali06@gmail.com (M.A.);; 2 MOE Joint International Research Laboratory of Animal Health and Food Safety, College of Veterinary Medicine, Nanjing Agricultural University, Nanjing 210095, China; 3 Department of Animal Nutrition, University of Veterinary and Animal Sciences, Lahore 54000, Pakistan

**Keywords:** *Cryptosporidium*, cryptosporidiosis, zoonosis, prevalence, food safety, One Health

## Abstract

A sharp rise in the global population and improved lifestyles has led to questions about the quality of both food and water. Among protozoan parasites, *Cryptosporidium* is of great importance in this regard. Hence, *Cryptosporidium*’s associated risk factors, its unique characteristics compared to other protozoan parasites, its zoonotic transmission, and associated economic losses in the public health and livestock sectors need to be focused on from a One Health perspective, including collaboration by experts from all three sectors. *Cryptosporidium*, being the fifth largest food threat, and the second largest cause of mortality in children under five years of age, is of great significance. The contamination of vegetables, fresh fruits, juices, unpasteurized raw milk, uncooked meat, and fish by *Cryptosporidium* oocysts occurs through infected food handlers, sewage-based contamination, agricultural effluents, infected animal manure being used as biofertilizer, etc., leading to severe foodborne outbreaks. The only Food and Drug Administration (FDA)-approved drug, Nitazoxanide (NTZ), provides inconsistent results in all groups of patients, and currently, there is no vaccine against it. The prime concerns of this review are to provide a deep insight into the *Cryptosporidium’s* global burden, associated water- and foodborne outbreaks, and some future perspectives in an attempt to effectively manage this protozoal disease. A thorough literature search was performed to organize the most relevant, latest, and quantified data, justifying the title. The estimation of its true burden, strategies to break the transmission pathways and life cycle of *Cryptosporidium,* and the search for vaccine targets through genome editing technology represent some future research perspectives.

## Simple Summary

*Cryptosporidium* is an emerging One Health concern that is equally important in the public health and livestock sectors. To fulfill the aims and objectives of this review, data regarding the unique characteristics of *Cryptosporidium*, its transmission pathways, its life cycle, its prevalence in both humans and animals from the perspective of its zoonotic potential, water- and foodborne outbreaks due to this protozoan parasite, and associated economic losses are briefly discussed, prioritizing the latest research and quantified data. Finally, some strategies for breaking transmission pathways and future research perspectives are discussed, to show the research gaps regarding this ever-increasing threat, which is of great scientific interest. Based on the estimation of global prevalence trends, transmission pathways, and overall burden in cryptosporidiosis, better managemental policies, surveillance programs, and preventive measures can be adopted for safeguarding, following the One Health approach.

## 1. Introduction

*Cryptosporidium* is a genus of protozoan parasites responsible for cryptosporidiosis. It was first identified in the early 20th century [1] and later attracted greater attention due to its increasing prevalence, wide host range, zoonotic potential, and unique characteristics, which have proven lethal in immunocompromised individuals [2]. It has been ranked fifth among twenty-four foodborne parasites worldwide [3]. Contaminated drinking and recreational water [4,5]; the consumption of a variety of vegetables [6], raw milk, meat, and apple cider juice [7]; and direct contact with infected food products can lead to an outbreak [8]. Of 22 foodborne outbreaks, Gharpure et al. calculated that 40.9% came from raw milk and 18.2% came from contaminated apple cider [5]. Also, the consumption of salad was detected in 35% of reports [9]. During 2010–2020, in 96.5% of *Cryptosporidium* food-related pathologies, *C. parvum* was detected as an etiological agent [10]. However, in the USA, about 50% of waterborne outbreaks were related to *C. hominis* [11]. In another outbreak, samples of goat milk tested positive via real-time PCR [12].

This parasite can infect a variety of vertebrates, e.g., fish, amphibians, reptiles, birds, and mammals [13], resulting not only in a huge economic loss to the veterinary sector [14] but also in its zoonotic transmission. For instance, waterborne outbreaks reported in 1993 in Milwaukee, Wisconsin, United States, were due to water contamination by grazing cattle and slaughterhouse effluents, affecting 400,000 individuals, with 69 deaths [15,16]. From 2011 to 2016, 63% of *Cryptosporidium*-related waterborne outbreaks lead to 4.2 million disability-adjusted life years (DALYs) [17], 69 million morbidities, and 57,203 mortalities [18]. From 2009 to 2017, over 444 outbreaks were reported in the USA. Investigations regarding these outbreaks showed that contact with infected cattle was responsible for 15% of outbreaks, while 12.8% of cases originated from contact with infected individuals in pediatric wards [5].

According to the Global Enteric Multicenter Study (GEMS), cryptosporidiosis is ranked as the second (after rotavirus) leading cause of moderate-to-severe diarrhea in infants aged 0–59 months, especially in Africa and Asia, responsible for approximately 202,000 deaths annually [19]. On average, *Cryptosporidium*’s prevalence is 4.3% and 10.4% in developed and developing countries, respectively [20]. Out of 47 *Cryptosporidium* spp. [21] and 120 genotypes [22], almost 21 species and 4 genotypes have been detected in humans, with *Cryptosporidium hominis* (*C. hominis*) and *Cryptosporidium parvum* (*C. parvum*) being the most prevalent among all [22,23].

In summary, cryptosporidiosis is an ever-increasing challenge for the food [3] and water industries [24], negatively influencing the public health and livestock sectors. So, giving immediate attention to this One Health threat of our time is vital. This article focuses on the life cycle and pathogenies of *Cryptosporidium* spp., its unique characteristics, associated risk factors, transmission pathways, the global impact of cryptosporidiosis, and food- and waterborne outbreaks, to provide readers with a deep understanding of the overall burden due to this zoonotic parasite, with the aim of establishing proper preventive and control strategies and finding potential therapeutics against it in the future.

## 2. Methodology

The information presented here was obtained by searching electronic internet databases like SCOPUS, Web of Science, PubMed, and Science Direct, as well as by searching Google Scholar and by visiting specific relevant journals. Various combinations of keywords, such as the global impact of *Cryptosporidium*, foodborne outbreaks, waterborne outbreaks, *Cryptosporidium* and One Health, zoonotic transmission, prevalence, and future perspective, were used. The review mainly included peer-reviewed scientific articles from world-renowned journals, written in English, focusing on the most recent and relevant data throughout the manuscript. Initially, 300 articles were downloaded; later, 68 articles were excluded due to low impact factors, their being old, and to avoid repetition.

## 3. Infectious Life Cycle and Pathogenesis of *Cryptosporidium*

Tyzzer initially characterized *Cryptosporidium* in 1907, but it remained largely unnoticed until the beginning of the 1980s, when it appeared as the root cause of chronic diarrhea and deaths in AIDS patients. The genus *Cryptosporidium* is currently classified into over 47 species with a broad host range including fish, amphibians, reptiles, birds, and mammals (157 mammalian species comprising, primates, Equidae, Carnivora, Bovidae, Leporidae, Rhinocerotidae, and Tapiridae) [25]. Although *C. hominis* and *C. parvum* account for over 90% of cases in humans [26,27], additional pathogenic species including *C. muris*, *C. andersoni*, *C. meleagridis*, *C. ubiquitum*, and *C. cuniculus* also show zoonotic potential [28].

**Figure 1:**
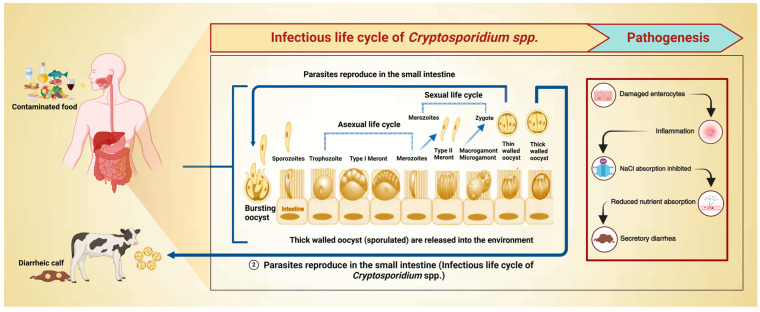
Infectious life cycle and pathogenesis of *Cryptosporidium* species. *Cryptosporidium* oocysts, after ingestion, complete their asexual and sexual life cycle in the epithelial cells. Upon the completion of the infectious life cycle, thin-walled oocysts usually cause autoinfection within the same host, while thick-walled oocysts are released into the environment and infect new susceptible hosts (e.g., calves).

The parasite’s life cycle culminates in the feces and releases contagious oocysts with thick outer walls [29]. Infectious oocysts contaminate grazing land, soil, and water. When oocysts are ingested by a susceptible host, interaction with acid in the stomach and bile salts along with the expression of surface receptors on oocysts and action of enzymes such as trypsin facilitate excystation resulting in the release of four motile sporozoites into the intestinal tract [30]. Secretary apical organelles are important for adherence, invasion, and insertion in host epithelial cells, subsequently, the parasite nestles itself inside a parasitophorous (intracellular but extracytoplasmic) vesicle for shelter and nutrition. The sporozoites take on an asexual reproduction cycle (merogony), featuring differentiation and, subsequently, trophozoite, Type I meront, and merozoite formation. Several of the merozoites penetrate the surrounding enterocytes (sustaining the asexual cycle), whilst others form Type II meronts (each with four merozoites). Type II merozoites then attach to neighboring host cells and develop into either microgamonts or macrogamonts, commencing gametogony (the sexual phase). Microgametes are formed after the maturation of microgamonts, which, after release, can fertilize the macrogamonts, generating a zygote. The zygote, after meiosis, develops into four naked sporozoites that later grow into oocysts, among which 20% of oocysts have thin walls, facilitating the host’s autoinfection. The remaining oocysts with thick walls (resistant to environmental stressors and promptly pathogenic) are discharged in the feces, generally in high proportions (Figure 1) [31].

The new generation of parasites can emerge and grow in just 12 to 14 hours [32]. The mean severity of infection by *Cryptosporidium* in cattle has been predicted to be around 2–1000 oocysts per gram of feces [33]. An infected cow can shed up to 40 kg of infected feces per day, with oocyst secretion persisting for a duration of 3 to 15 days in infected animals. Humans are also significant contributors to the ecosystem’s oocyst pool (105–107 oocysts/g), which causes surface water contamination [10].

The incubation period typically spans from 3 to 15 days; in the majority of cases, clinical manifestations start from 5 to 7 days and can persist for up to one month [34]. Host cell invasion is limited to the luminal border, deterioration of epithelial cells, metaplasia of villi, hyperplasia of crypt epithelium, and dislocation of microvilli, and lengthy microvilli can be observed close to the parasitic stage [35]. Damage to tissues stimulates the host’s immunological reactions, resulting in the recruitment of prostaglandin-secreting inflammatory cells. Crypt cells and prostaglandins promote chloride ion release, which reduces NaCl absorption. Secretory diarrhea develops as a result of poor intestinal fluid and food absorption, as well as disruptions to normal ion flux. Extra-gastrointestinal cryptosporidiosis has been described in both immune-competent and immunocompromised individuals, infecting the pancreas, liver, and bile ducts. Extreme cases have included tracheobronchial involvement and even sinusitis. The particular pathogenic processes of cryptosporidiosis have yet to be fully understood; however, in vitro investigations with Caco-2 cells have shown that enterotoxins may be associated with the procedure, as reported. The most common long-term symptoms are diarrhea, abdominal discomfort, vomiting, exhaustion, and migraine [36]. In immunocompetent people, the signs of cryptosporidiosis infection are typically self-limited, whereas, in immune-compromised patients, the gastrointestinal infection is chronic and persistent and can influence atypical and extra-intestinal regions [37].

## 4. Potential Risk Factors Fostering *Cryptosporidium* Spread

**Figure 2:**
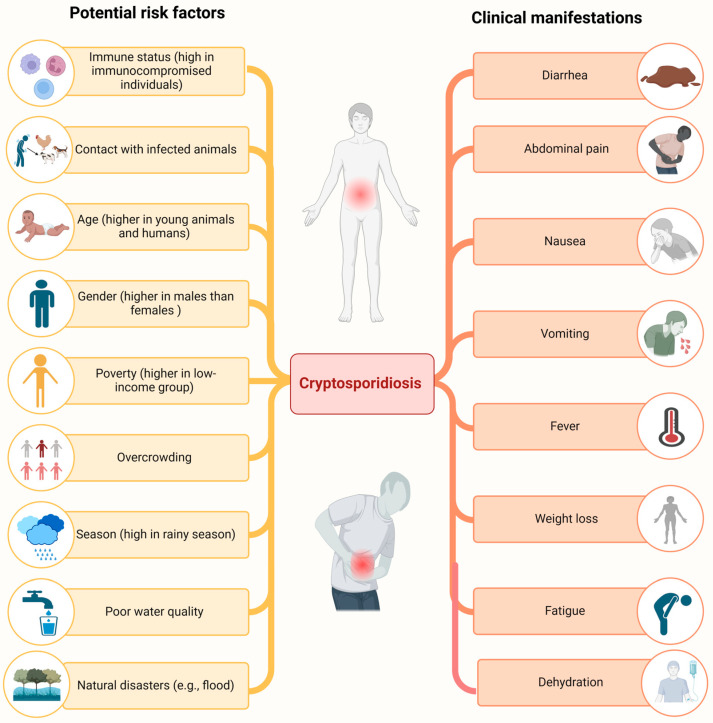
Risk factors and clinical manifestations associated with cryptosporidiosis. Risk factors like immune status, sanitary conditions, age, natural disasters, etc., lead to *Cryptosporidium*-associated outbreaks. The disease is mainly manifested by secretory diarrhea and other consequences related to gastrointestinal tract disturbances.

The spread of *Cryptosporidium* infection is linked to interactions with contaminated animals, age (the infection rate is greater among young animals, children, and humans over 75 years old), gender (the infection rate is greater in men), poverty (poor sanitary facilities and water quality), illiteracy, sexual exposure (anal) to human feces, high population density, seasonal variations (an increase in cases is observed during late summer and monsoon (often associated with swimming in contaminated water)), immune status of an individual e.g. HIV-positive patients with CD4+ lymphocyte counts of <50/mm^3^ [38] and patients who have undergone organ transplant surgeries are more susceptible [39]. Similarly, other risk factors include occupational activities (veterinarians, vaccinators, pet owners, hunters, etc.), natural disasters (windstorms, earth erosion, or floods), a shift from endemic to vulnerable areas, increasing temperatures, a lack of infrastructure growth, and social and political disputes (Figure 2) [24,40].

Seasonal variations in the occurrence and quantity of *Cryptosporidium* spp. oocysts depend on the temperature, parasitic species, and the source of water (farming, debris, domestic, or runoff water) [41]. One possible explanation for such fluctuations in the prevalence of intestinal infections is the source of contamination and the mechanisms that allow pollutants’ infiltration into aquatic ecosystems. For example, enteric infections of zoonotic importance are largely discovered during storms, excessive rain, and grazing periods post-snowmelt and are associated with agricultural effluents [42], resulting in numerous outbreaks. Hence, the spring peak may indicate livestock activities (e.g., calving), as well as an increased risk of water spoilage due to prolonged rainfall or snow thawing [43]. In other cases, the late-summer surge may indicate visitors returning home after visiting more prevalent locations, as well as behaviors that raise exposure dangers, such as increased contact with recreational water and changes in food consumption behaviors, promoting the unintentional ingestion of oocysts [44].

## 5. Unique Characteristics of *Cryptosporidium*: A Challenge in Tackling *Cryptosporidium*

Current preventive measures and available therapeutics are not fully effective against *Cryptosporidium* [45,46] due to its distinct characteristics, which include its unique locations inside the host cell, the presence of the parasitophorous vacuole, the presence of thick (cause new infection in the susceptible host through environmental spread) and thin-walled (responsible for autoinfections) oocysts, as well as tiny size of oocysts (4 to 9 μm), the absence of specific morphological traits such as sporocysts or micropales, reduced susceptibility to all available anticoccidial medications [47], the oocysts’ resistance to disinfectants, e.g., chlorine [48], and the ability of oocysts to persist in surface water and soil for up to two years due to their resilient outer wall composed of a dual covering of a lipid–protein–carbohydrate complex [49]. Although ultraviolet irradiation can render oocysts non-pathogenic [50], they are excreted in very high quantities, particularly by young calves [51]. The minimal infectious dosage ranges from 10 to 100 oocysts [52]. Additionally, the oocysts’ survival on vegetables and fruits is not altered by low temperatures over days to weeks in a refrigerator [53]. *C. parvum* oocysts can also thrive at −20 °C for longer durations in cryoprotectants [54], with a prolonged incubation period ranging from 7 to 15 days [37], which hinders the detection of their origin and the execution of measures to stop their spread and continuous shedding in feces for up to 2 months after the relief of digestive symptoms. However, research studies investigating the elimination of *Cryptosporidium* oocysts from intentionally contaminated raspberries and blueberries revealed that rinsing in a vinegar solution increased the infectious pathogen removal efficiency by over 90% [55].

## 6. Zoonotic Transmission of *Cryptosporidium*: A Growing Public Health Threat

**Figure 3:**
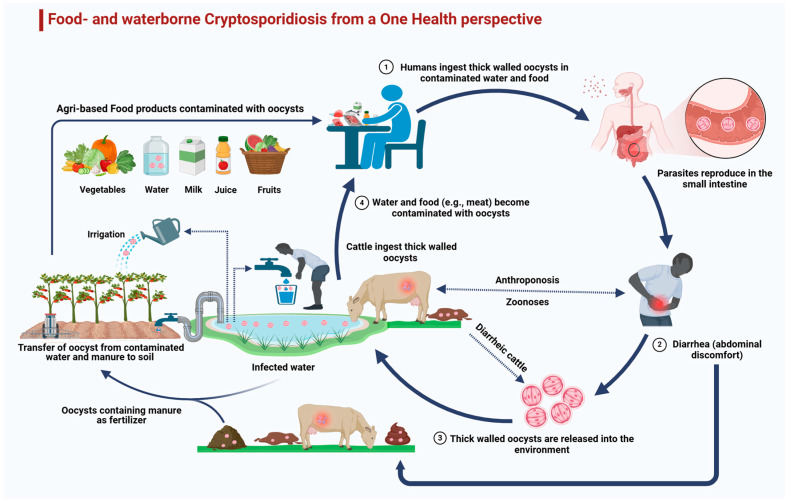
A One Health perspective on *Cryptosporidium* transmission, environmental contamination, and zoonotic transmission between humans and livestock. After completing their life cycle, oocysts contaminate the environment and reach livestock (anthoponoses), and from animal dung, oocysts reach the soil, contaminate water bodies, and can directly or indirectly contaminate food items and ultimately reach humans (zoonoses). This cycle continues between humans and animals, in which the environment acts as a medium.

Humans are sensitive to a broad diversity of *Cryptosporidium* spp. and genotypes (Table 1), particularly *C. parvum* and *C. hominis* [56,57]. *C. parvum* has an extremely wide host range, affecting fish, ungulates, rodents, carnivores, non-human primates, and mammals in aquatic ecosystems, among others, and a significant zoonotic potential in human beings due to common interactions with livestock species [10,58,59]. Two investigations in China indicated that *C. andersoni* showed the highest prevalent *Cryptosporidium* spp. found in humans [60,61]. However, further investigation is needed to properly understand the zoonotic relevance of *C. andersoni*. Multiple studies have detected *C. bovis* in humans (Table 1). *C. bovis* was initially diagnosed in an animal handler from India in 2010 [62]. It was also detected in a 3-year-old toddler and a 23-year-old adult from two different farms in Australia in 2012 [63]. Infected individuals consumed raw milk and had intensive interaction with dairy animals [63]. Another study investigated a combined *C. bovis* and *C. parvum* infection in a diarrheic young child aged less than 6 years from Egypt, who had contact with cattle, resulting in mixed infections [64]. Hence, the potential of cattle to transmit several species of *Cryptosporidium*, particularly *C. hominis*, represents a major public health hazard associated with possible interactions (Figure 3) [65].

*Cryptosporidium scrofarum* (*C. scrofarum*) and *C. suis* are the prevalent species infecting pigs, but *C. suis* has also been detected in HIV-positive individuals in Thailand, Cambodia, China, and Peru [61,66,67], and *C. suis*-positive patients have been detected in Madagascar and the United Kingdom (UK) (Table 1) [68]. There is another report of *C. scrofarum* in a 29-year-old man co-infected with *Giardia* [69]. There are also rare cases in which infection transmission occurs from pets to humans [70]. Further research findings suggest that 84% of all *Cryptosporidium* infections are locally obtained, induced by *C. parvum* [71]. In the Netherlands in 2017, cryptosporidiosis was valued at EUR 19.2 million. In accordance with the mentioned study, the total illness burden owing to *Cryptosporidium* infestation was 3.14 × 10^−5^ and 9.68 × 10^−6^ DALYs per person per year for cancer patients and immunocompetent individuals, respectively [72].

**Table 1:** *Cryptosporidium* species, associated hosts, zoonotic spread, and site of incubation.

Species	Main Host	Zoonotic Potential	Oocyst Count (μm)	Location	Reference
*C. andersoni*	Cattle, dromedary and Bactrian camels, mice, sheep, wood partridges	Yes	N/A	Gastric localization	[73]
*C. baileyi*	Birds	No	6.4 × 6.2	Cloaca, bursa, respiratory tract	[74,75]
*C. cuniculus*	European rabbits	No	5.98 × 5.38	Small intestine	[76]
*C. erinacei*	Hedgehogs, horses	Yes	4.9 × 4.4	Small intestine	[77]
*C. fayeri*	Kangaroos	Yes	4.9 × 4.3	Small and large intestine	[78]
*C. galli*	Wild birds (goldfinches, green-winged saltators, saffron finches)	Yes	8.0–8.5 × 6.2–6.4	Small intestine and caecum	[79]
*C. muris*	Rodents	Yes	5.6 × 7.4	Stomach	[80]
*C. occultus*	Rats	Yes	5.20 × 4.94	Colon	[81]
*C. ornithophilus*	Birds: geese, chickens, and cockatiels	No	6.13 × 5.15	Caecum, colon bursa of Fabricius	[82]
*C. parvum*	Alpacas, cattle, red deer, goats, sheep	Yes	4.5 × 5.5	Small intestine	[73]
*C. proventriculi*	Psittaciformes birds	No	7.4 × 5.8	Proventriculus	[13,74]
*C. rubeyi*	California ground squirrels	No	4.4 × 4.34	Large intestine	[83]
*C. ryanae*	Ruminants	No	3.2 × 3.7	Small intestine	[84,85]
*C. saurophilum*	Lizards, snakes	No	4.2–5.2 × 4.4–5.6	Small intestine and cloaca	[86]
*C. suis*	Pigs	Yes	5.1 × 4.4	Small intestine	[87]
*C. suis*	Pigs	Yes	4.6 × 4.2	Small and large intestine	
*C. tyzzeri*	Mice and other rodents	Yes	4.64 × 4.19	Small intestine (jejunum and ileum)	[88]
*C. ubiquitum*	Cattle and goats	Yes	5.04 × 4.66	Small intestine	[89]
*C. wrairi*	Guinea pigs	Yes	4.0–5.0 × 4.8–5.6	Small intestine	[90]
*C. xiaoi*	Sheep and goats	Yes	5.24 × 4.79	Small intestine	[89,91]
*C. meleagridis*, *C. baileyi*	Chickens, turkeys, quails	Yes	4.5–5.0 × 4.6–5.2	Small and large intestine and bursa	[92]
*C. galli, C. baileyi*, and *C. meleagridis*	Pet birds (Rufous turtle dove, white Java sparrows, white-tailed pigeons)	Yes	5.8 × 4.5	Small intestine	[93]

N/A, Not Available (The exact oocysts count is not available).

## 7. Prevalence of *Cryptosporidium* in the Livestock Sector, Contamination Sources, and Exposure Routes

The prevalence of *Cryptosporidium* was found to be 27.8–60.4% in pigs, 18% in calves, 52.7% in dogs, 28.52% in cattle, and 29.4% in cats [65,94]. Regarding cattle, the major species are *C. bovis*, *C. andersoni*, *C. parvum*, and *C. ryanae*; although, *C. scrofarum*, *C. hominis*, *C. occultus*, *C. felis*, *C. suis*, *C. serpent*, *C. meleagridis,* and *C. tyzzeri* have also been reported [10,59]. However, in calves, the likelihood of *Cryptosporidium* infection is influenced by age. *C. parvum* infects calves up to 8 weeks old, while *C. bovis* is prominent in calves aged 3 to 11 months [95]. Research in Norway found that approximately 50% of the dairy farms had calves infected with *C. parvum* [96]. In Henan (China), two large-scale research studies reported that calves before weaning were found to be infected with *C. bovis* rather than *C. parvum* [97]. *C. bovis* commonly causes infection in pre-weaned calves, which are typically retained under conventional animal management systems [98]. *C. xiaoi*, *C. parvum*, and *C. ubiquitum* are the predominant species in goats and sheep; however, *C. occultus*, *C. andersoni*, *C. baileyi*, *C. ryanae*, *C. hominis*, *C. suis*, *C. muris*, *C. fayeri*, *C. bovis*, *C. scrofarum*, and *C. canis* have also been also documented in small ruminants [10,58,59]. In China, *C. hominis* has been detected as a major *Cryptosporidium* spp. among donkeys and horses [99].

Several molecular epidemiologic investigations into *Cryptosporidium* spp. in pigs and other species have been reported. In China, Eurasian wild boars were found to be infected with *C. scrofarum* (Table 2) [100]. *C. andersoni* has been detected in giant pandas [101] and in cows, goats, and sheep [58] and has been documented in deer [102] and rodents, especially hamsters [103]. Surprisingly, in China, *C. ubiquitum* was diagnosed in hedgehogs and chinchillas [104]. *C. hominis* has been reported in fish, flying foxes, rodents, non-human primates, marsupials, cattle, Bactrian camels, sheep, goats, donkeys, horses, dingoes, foxes, badgers, birds, dugongs, and clinical laboratory infections have been developed in mice, gerbils, lambs, calves, and piglets [105]. In New Zealand, it was found that 80% of calves were detected to have *C. hominis* infection [106], while, in South Korea, *C. hominis* was the sole species detected in goats [107]. These investigations show that goats, sheep, and cattle have the potential to function as animal reservoirs for *C. hominis* infestations [59,106]. In addition to sheep and cattle, *C. hominis* has been observed in marsupials, deer, and dingoes that inhabit areas with drinking water spills [106,107,108]. It is certain that this came from human spill-back through wastewater contamination in grasslands or direct contact. Furthermore, in the central Sichuan Province, fecal analysis on 278 pre-weaned dairy calves revealed that 40 (14.4%) calves were found to be positive for *Cryptosporidium* infection, with 28 infected with *C. bovis*, 7 infected with *C. parvum*, and 5 infected with *C. ryanae* [109]. The prevalence of *Cryptosporidium* spp. was around 4.7% (16/342; *C. xiaoi* (11/16) and *C. suis* (5/16)) in goats in the Sichuan Province [110]. This shows the circulation of *Cryptosporidium* between humans and animals.

## 8. Cryptosporidiosis: Waterborne and Foodborne Outbreaks and Their Consequences

### 8.1. Presence of Cryptosporidium in Water Bodies and Associated Waterborne Outbreaks

In the last decade, the global population has more than tripled, and water requirements have increased fourfold, resulting in an elevated need for water reserves globally [124]. As well as there being a scarcity of water, the purity of water is uncertain. Almost 2 billion people utilize polluted water resources, culminating in around 485,000 deaths/year from diarrheal infections [125]. Waterborne protozoal infections pose a global health problem with worldwide dispersion, and they have been recorded in both developed and underdeveloped nations with a consistently rising incidence of outbreaks [126,127,128]. The CDC estimated a twofold rise in the proportion of waterborne outbreaks caused by *Cryptosporidium* from 2014 to 2017 [129]. For instance, from the beginning of the past decade to 2016, cryptosporidiosis was accountable for 60% of all documented waterborne epidemics induced by protozoan parasites [126,128].

As diseased animals and humans can spread enormous quantities of transmissible oocysts in the environment, this pathogenic organism has been detected globally in rivers, lakes, and drinking water samples. Furthermore, oocysts may contaminate underground water reserves by infiltrating polluted water surfaces. Usually, documented oocyst concentrations typically range from 0.01 to 150 oocysts per liter, but larger quantities have been detected in farming run-off and residential water waste materials. In 1993, a significant waterborne disease occurred in Milwaukee, Wisconsin (USA) [130]; the total cost was USD 96.2 million, including medical expenditures of around USD 31.7 million, and production losses accounted for USD 64.6 million [131]. To date, at least 165 *Cryptosporidium* waterborne outbreaks have been reported in the developed world with a greater proportion stemming from Europe and North America [126,132,133]. In Ostersund, Sweden, the second greatest waterborne epidemic emerged, infecting 27,000 people in 2010 [7]; a similar waterborne infection impacted around 18,500 residents of Skellefteå in 2011. The worldwide waterborne outbreak framework regarding human cryptosporidiosis indicates that its prevalence in emerging economies will drop by 24.5% in 2050; although, in Africa, exposure to freshwater bodies will rise by over 70% (Table 3) [134].

An examination of 325 cases of human infection due to the aquatic spread of pathogenic organisms revealed that more than 51% were triggered by *Cryptosporidium* infection [126]. A total of 22.5% of samples taken from the Han River in Korea were identified as positive for *Cryptosporidium* oocysts [135]. The average abundance of *Cryptosporidium* in North America and Middle East water bodies was identified as 24.5% [19]. The worldwide prevalence reported for waterborne cryptosporidiosis cases appears to be weighted significantly towards North America, with a minimum of 40% of cases reported up to 2010 coming from Canada and the USA; the proportion of reported infection incidents in the UK was 77% (Table 3) [126,128]. This imbalanced proportion for the UK and North America indicates better surveillance, inspection, and reporting processes.

Of the 325 water-linked incidents of parasitic infections reported globally [126], *Cryptosporidium* contributed to 50.8%, with 50.3% linked to contaminated pool water and 23.7% linked to contaminated water distribution systems [24]. Consequently, the WHO set a recommendation of one *Cryptosporidium* oocyst/1.600 L of drinking water to reach the health outcome aim of 106 DALY pppy [136].

**Table 3:** Waterborne outbreaks caused by *Cryptosporidium* species in humans.

Country (Year)	No. of Cases	Age Group	Source of Contamination	Diagnostic Technique	Species and Subtype of *Cryptosporidium*	Case-Associated Details	Reference
Denmark (2022 to 2023)	7	N/A	Drinking water	Sanger sequencing to detect the species, and more specific gp60-based PCR*	*C. ditrichi*, *C. tyzzeri*, *C. viatorum*, and *C. mortiferum * XIVaA20G2T1	Identifying causes of gastrointestinal symptoms was linked with traveling outside Denmark.	[137]
Finland (2019)	272	Adults	Water source and contact with infected cattle	ZN staining*, RT-PCR* method for rRNA gene of *C. parvum*	*C. parvum* and * C. hominis * IIaA15G2R1, IIaA13G2R1	A total of 65% of cases had regular cattle contact due to work or studies. The most common symptoms were diarrhea in 97% of patients, weakness in 83%, and stomachache in 76%; the symptomatic period was 12 days.	[138]
France (2017)	100	Adults	Groundwater	RT-PCR* by GP60 sequencing	*C. hominis* *IbA10G2*	The attack rate was 27.8%; abdominal pain was identified in 84% of cases, diarrhea in 68%, nausea in 53%, and fever in 46%.	[139]
France (2017–2019)	443	Children and adults (aged < 75 years)	Direct and indirect water contact	N/A	*C. Parvum* and *C. hominis* IbA10G2, IaA22R2	Diarrhea was reported in 20% of cases; a higher proportion of females were infected with *C. hominis* (55%) compared to those infected with *C. parvum* (45%).	[140]
Italy (2019)	71	Children and adults	Water supply	PCR* and sequencing of the small subunit rDNA	*C. parvum* *IIdA25G1*	Overall, 79% of cases tested positive for infection.	[141]
Spain (2023)	365	Young children	Swimming pool and drinking water	Multi-target PCR* panels and microscopic examination	*C. parvum* and * C. hominis * IfA12G1R5	*C. hominis* was isolated from 94.3% of the case samples and *C. parvum* from 5.7% of cases.	[142]
Spain (2018)	25	Children	Splash pad	Microscopy analysis	*C. parvum*	Overall prevalence was 35.2%; diarrhea was reported in 97.2% of cases, followed by abdominal pain in 71.8%, nausea in 29.6%, and fever in 19.7%.	[143]
Spain (2013)	7	Children (aged < 4 years)	Drinking water	nPCR* analysis of the SSU* rRNA PCR gene	*C. hominis* IaA11R2 and IbA10G2	Major symptoms were acute gastroenteritis, diarrhea, and abdominal pain.	[144]
Sweden (2013–2014)	398	Children and adults (aged 1 to 88 years)	Food and drinking water	ZN staining*, RT-PCR*, molecular analysis, RFLP analysis	*C. parvum* IIaA16G1R1, IIaA15G2R1, and IIdA20G1	A total of 63% of cases were reported in Sweden; among them, 55% were women and 45% were men.	[71]
Sweden (2018)	21	Children and adults	Drinking water and red squirrels	Amplification of the SSU* rRNA gene by PCR*	*C. chipmunk* and * C. parvum * XIVaA20G2T1e	Major cases appeared from August to December, initially, thought to be food item-related but not confirmed.	[145]
Sweden (2010–2011)	753	Children (aged <15 years)	Water treatment plant	Microscopic examination and nPCR*	*C. hominis*	The attack rate was 43.6% in children who consumed contaminated water, and diarrhea was reported in 82% of cases.	[146]
UK (2022)	250	All ages	Tap water	ELISA	*C. parvum*	Due to heavy rain, contamination from agricultural runoff was detected.	[147]
China (2016)	400	Children and adults (aged 7 months to 89 years)	Wastewater	nPCR* amplifies the gene encoding SSU* rRNA	*C. viatorum*, *C. occultus*, and *C. viatorum* eXVaA3h	Males accounted for 48.8% of infections, and females accounted for 51.2% of infections, with a prevalence rate of 0.5%.	[148]
China (2021)	684	Children	Water and animal contact	Molecular identification, DNA extraction, and genotyping	*C. parvum*, * C. baileyi,* *C. viatorum* *XVaA3*, * C. felis XIXa*, and *C. parvum* *IIdA19G1*, * IInA10*	The overall prevalence rate was 21.3%; *C. felis* was only reported in patients with diarrhea, and *C. baileyi* was documented in asymptomatic people.	[149]
Kuwait (2000–2008)	2548	Children (aged < 16 years)	Overhead water tank	Microscopic examination, PCR*, nPCR*, RFLP* analysis, and DNA sequencing	*C. parvum * IIa	A total of 41.4% prevalence was found in the 4-to-8-year age group compared with the other age groups.	[150]
Turkey (2021)	69	Adults and children	Drinking water	Acid-fast staining and nPCR*	*Cryptosporidium* spp.	*Cryptosporidium* infection was identified in 10.1% of cases; the prevalence of parasitic agents in drinking water will be high if more research studies are conducted in Turkey.	[151]
Sudan (2020)	150	Children (aged < 5 years)	Water sources from pipe canals and donkey cart	ZN staining* technique and microscopic examinations	*C. parvum*	An overall prevalence of 27.1% was observed in children with diarrhea but lower prevalence was observed in children without diarrhea (8.8%).	[152]
Nigeria (2019)	500	Infants (up to 12 month)	Contaminated water	Microscopy	*C. hominis*	A high incidence in rural areas with an increased mortality rate was observed due to limited access to clean water.	[153]
South Africa (2020)	400	Children (1–5 years)	Recreational water	PCR	*C. hominis*	Swimming pool-related outbreaks in summer holidays were detected.	[154]
Canada (2016–2017)	201	Children and adults (aged < 34 years)	Water amusement park and swimming pool	nPCR* based amplification and sequencing of the gene encoding SSU* rRNA	*C*. *parvum* IIaA15G2R1, IIaA16G3R1, and IIaA16G2R1	In total, 23% of cases were infected with *C. hominis* while 74% were infected with *C. parvum*; the average annual incidence rate was higher for females (20 to 34 years old) compared to males.	[155]
USA (2015–2020)	1200	All ages	Recreational water	PCR, microscopy, IFAT	*C. hominis*	Outbreaks were associated with public pools, with a significant increase in cases during summer.	[156]
South America (2018)	16	Children and adults	Maroni river and ground water	ZN staining* and RT-PCR*	*C. hominis* *IbA10G2* and * C. parvum* *IIdA19G2*	Major symptoms of gastrointestinal illness included diarrhea, nausea, and abdominal pain.	[157]

PCR*, polymerase chain reaction; ZN staining*, Ziehl–Neelsen staining; nPCR*, nested polymerase chain reaction; RT-PCR*, real-time polymerase chain reaction; RFLP*, restriction fragment length polymorphism; SSU*, small subunit. N/A, Not Available (The exact oocysts count is not available).

### 8.2. Environmental Circulation of Cryptosporidium: Implications for Foodborne Outbreaks

On a global scale, *Cryptosporidium* has been classified as the 5th most significant disease out of 24 foodborne parasitic infections, with only *Echinococcus granulosus*, *Toxoplasma gondii*, *Echinococcus multilocularis*, and *Taenia solium* surpassing it [3], and has induced 8.6 million incidents of foodborne disease in 2010, that comprised 3759 deaths of individuals and 296,156 DALYs [6]. *Cryptosporidium* is among the top 10 foodborne diseases identified by the FoodNet monitoring program in the US [158]; a European classification of foodborne pathogens [159] also listed *Cryptosporidium* as the second most significant foodborne disease in Western and Northern Europe [160]. Food exposure with *Cryptosporidium* oocysts may originate during manufacturing, and oocysts are harbored on wet surfaces of vegetables and fruits [9]. Previous Swedish foodborne epidemics have been induced mostly by *C. parvum* and *C. hominis* connected with the ingestion of contaminated arugula salad [161], parsley [162], and unprocessed spinach juice [163]. However, the cause of contamination cannot be identified in any of these cases.

**Figure 4:**
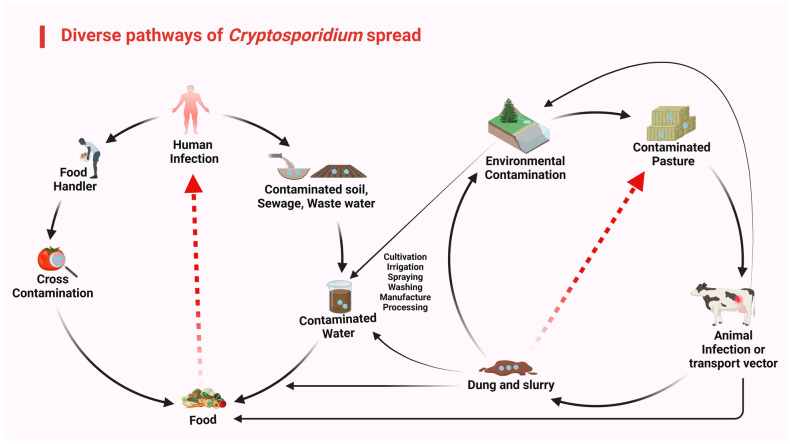
Diverse pathways for the spread of *Cryptosporidium* oocysts. This figure shows different transmission routes of *Cryptosporidium* oocysts (shown by arrows) between humans and animals involving different agricultural practices. By breaking transmission routes, outbreaks can be prevented.

The agriculture sector contributes 70% of global water resources; consequently, farm-based effluents lead to the contamination and destruction of the natural ecosystem [164,165]. In Europe, 38% of water reservoirs are endangered by industrial contaminants [166]. In developing countries, industrial pollutants have also been considered an issue due to rising debris runoff [166]. WHO and FAO assessments advise the consumption of at least 400 g of daily vegetables and fruits to prevent chronic infections and also to avoid a deficiency in micronutrients [167], but these become infected due to the cultivation of crops with fecal-infected water, the use of animal dung on crops as a natural fertilizer (cattle produce 3.2 × 10^23^ oocysts/year), and the use of infected water to dilute chemicals (Figure 4) [168]. Spreading is also enhanced by the possibility that *Cryptosporidium* oocysts may persist within, and be shielded by, the stoma of leafy vegetables and fruits [169]. Wildlife, especially wild birds, ruminants, and boars, may also be transmitters of *C. parvum* and could harm the environment, agricultural products, and water [170]. Infectious agents can also be waterborne or transmitted through human handling of fresh products [171].

Several foodborne outbreaks related to contamination from cattle waste have been documented. Six percent of fruits and vegetables were found positive for *Cryptosporidium* in Norway. The vegetable samples comprised radish sprouts, strawberries, lettuce, dill, and mung bean sprouts [172]. A similar study carried out in Poland using six vegetable samples, which were gathered from an area of significant animal production, confirmed the presence of *Cryptosporidium* oocysts. Also, another outbreak of *C. parvum* by the consumption of food was thought to have been spread by the ingestion of vegetables that had been sprinkled with water that was probably contaminated with animal dung [173].

Fermented milk, salads, raw vegetables, raw milk, beef, and apple cider are among the main food items where the existence of oocysts has been observed. The two waterborne epidemics reported in Europe in 2010–2011 affected 47,000 people in Sweden [7]. Unprocessed raw milk has also been involved in foodborne outbreaks of cryptosporidiosis [174]. There is no proliferation of *Cryptosporidium* spp. in milk, but infectious oocysts can persist there. A research study in Iraq indicated that the prevalence of oocyst detection in raw caprine and ovine milk samples was 46% and 32%, respectively [14]. Loury et al. (2019) identified that infection was related to the eating of uncooked meat; although, in other infections, dairy products and unpasteurized milk appeared as a risk factor [175]. Another study reported an outbreak in 2003 in the USA in which apple cider vinegar was the cause of transmission of *C. parvum* GP60 subtype IIaA14G1R1 for only one patient, while *C. ubiquitum* was found to be responsible for other cases [176]; this outbreak was investigated to be originated from a container of self-pressed apple juice [177]. In the USA, roughly 10% of all cases of cryptosporidiosis are reported to be foodborne [178].

In the western region of the Korean Peninsula, reports have shown the contamination of 32.4% of environmental soil samples and 12.5% of vegetable samples with *C. parvum* [179], and in another research study by Korean scientists, it was documented that *Cryptosporidium* was detected in 7.7% unprocessed vegetables [6]. Domenech et al. (2018) [180] stated that the *Cryptosporidium* oocyst count ranges from 1.38 to 2.6/L in processed wastewater utilized for the irrigation of leafy green vegetables. In Henan Province of China, Li et al. (2019) analyzed 21 fruits and 1099 vegetable samples and reported that *C. parvum* was only found in 1% of the fruits and vegetables [181]. *Cryptosporidium* spp. were identified in 2.6% of the herbs and vegetables taken directly from fields and supermarkets in the Czech Republic [182]; also, *Cryptosporidium* spp. were documented in 11.3% of vegetables in Brazil [183]. In addition, *Cryptosporidium* oocysts were reported in 6% of fresh vegetables in Chandigarh, India [184]. In Italy, unexpectedly, *Cryptosporidium* spp. was identified in 0.9% of packaged salads [185]. In Tehran, the documented prevalence rate of *Cryptosporidium* in vegetables was 6.65% (Table 4) [186].

*Cryptosporidium* oocysts were detected in 5.9% of samples of packaged leafy greens, including radicchio, endive, escarole, iceberg lettuce, baby lettuce, and romaine lettuce, in Canada [187]. In Zaria metropolis, Kaduna State, Nigeria, among 200 vegetables detected by modified Ziehl–Neelsen staining and sucrose flotation technique, 35% were found to be positive for *Cryptosporidium*. The peak contamination was recorded for lettuce (48%), while root vegetables (24%) had the lowest contamination rate. Additionally, 30% of raw vegetables and 40% of those that were cooked were found to be positive [188]. El Sherbini et al. (2016) [189] examined 10 types of fruits and vegetables in Egypt, including pepper, cabbage, tomato, carrot, lettuce, parsley, watercress, onion, and leek, and found that 18.1% were contaminated with *Cryptosporidium*, with the highest contamination rate in lettuce (26.3%), followed by leek (22%) [189]. In another study in Egypt, *Cryptosporidium* was identified in the vegetable samples, and the recorded prevalence rate was 29.3% (Table 4) [190].

In Denmark, almost 75 cases of intestinal inflammation due to *C. hominis* were identified, and 13% were in those who consumed salad contaminated by infected food handlers in a cafeteria [191]. Another outbreak was reported to be caused by the consumption of edible herbs, and *Cryptosporidium* oocytes were found in 30 fecal samples [192]. These findings highlight the need for more combined efforts against infectious pathogens and also reinforce the *Cryptosporidium* surveillance strategy for food matrices to avoid further outbreaks [193].

**Table 4:** Foodborne outbreaks due to *Cryptosporidium* in humans.

Country (Year)	No. of Cases	Age Group	Source of Contamination	Diagnostic Technique	Species and Subtype of *Cryptosporidium*	Case-Related Details	Reference
Finland (2012)	250	Children and adults	Ready-to-eat vegetables (Frisée salad)	nPCR*, microscopic examination, and antigen detection tests	*C. parvum * IIdA17G1	Symptoms included diarrhea and stomach pain; the outbreaks occurred in five lunch restaurants.	[194]
Italy (2019)	72	Adults	Packaged salads and fruit	Microscopic examination and PCR*	*C. ryanae*, * C. bovis*, * C. xiaoi*, and * C. ubiquitum*	The overall prevalence was 5.1%. Findings demonstrated the improper handling of fresh products, both locally manufactured and imported.	[195]
Norway (2018)	6	Adults	Self-pressed apple juice	nPCR*, target SSU-rRNA loci, and GP60	*C. parvum* IIaA14G1R1	The incubation period lasted 1 week, with symptoms persisted for up to 3 weeks.	[177]
Poland (2006–2007)	128	Adults and children	Fresh vegetables and soft fruits	IFAT* and microscopic examination	*C. parvum*	Overall contamination was 3.6%, with oocysts ranging from 1 to 17 in leek, red cabbage, and celery.	[196]
Romania (2022–2024)	426 (215 males and 211 females)	Children and adults (ages ranged between 1 and 93 years)	Consumption of uncooked meat and unhygienic fruits and vegetables	One-step *Cryptosporidium* card test and microscopic examination by the flotation technique	*C. parvum* AF108861, AF115377, CP141124, CP082119, OL348153, MN047127, and KT151531	The overall prevalence of gastrointestinal parasites was 3.99%.	[197]
Sweden (2019)	122	Children and adults (11 to 79 years)	Spinach juice	N/A	*C. parvum* IIdA22G1c and IIdA24G1	More women (62%) than men (38%) became ill.	[198]
Sweden (August, 2016)	7	Adults	Romaine lettuce, chicken, parmesan, spinach pie, and feta	PCR*, amplicon, shotgun sequencing, and molecular identification by the amplification of the 18S rRNA gene	*C. parvum*	The incubation period lasted 7 days, with watery diarrhea being the main symptom.	[199]
Sweden (2010)	27	Adults (<50) years)	Meals at restaurants	PCR* and RFLP*	*C. felis*, * C. hominis* and *C. parvum* IIdA24G1, and IIdA20G1e	Females accounted for 65% of cases, and diarrhea was present in all cases; although, bloating was reported in 91% of cases, and abdominal pain in 78% of cases.	[192]
UK (2012)	648	Adults (under 50 years old)	Ready-to-eat, precut mixed salad leaves	Microbial analysis and PCR	*C*. *parvum* IIaA15G2R1	A totel of 78% of cases were reported with subtype IIaA15G2R1; no proof was found for a mixed infection of *C*. *parvum* and *C. hominis*.	[200]
Nigeria (March, 2023)	384	Adults	Consumption of raw vegetables	The use of floatation, sedimentation, ZN staining techniques, and microscopy	*Cryptosporidium* spp.	The overall prevalence of oocysts in raw vegetables was 7%; cabbage had the highest number at 12.5%, followed by tomato at 11.3%, and the lowest number was recorded in garden eggs, at 1.2%.	[201]
Nigeria (2023)	500	Infants under 1 year	Contaminated vegetables	PCR	*C. hominis*	A high incidence was detected in villages associated with washing vegetables with contaminated water.	[153]
China (2023)	150	Adults (18–65 years)	Raw milk	PCR	*C. parvum*	The urban area was affected mostly due to the consumption of unpasteurized dairy products.	[202]
China (2021–2022)	2435	Children	Food	Microscopic examination and molecular identification by targeting 18S rRNA gene sequence	*C. parvum * IIdA23G3, IIdA24G3, IIdA24G4, IIdA25G3, and IIdA25G4	The overall prevalence was found to be 12.85%; fecal samples were collected from Inner Mongolia Maternal and Child Health Care Hospital.	[203]
USA (2014)	11	Children and adults (2 months–76 years)	Unpasteurized goat milk	N/A	*C. parvum,* IIaA16G3R1	The most severe cases were reported in those who drank raw goat milk, and three cases were identified among those who had contact with individuals who consumed the raw goat milk.	[204]
USA (2016)	7	Adults	Unpasteurized milk from a local New Mexico dairy	PCR*, microscopic examination, RFLP* and DNA sequencing	*C. parvum* IIaA18G3R	Cryptosporidiosis was typically linked to recreational water.	[205]
USA (2017)	1200	All ages	Raw milk	PCR*, microscopic examination, RFLP*	*C. parvum*	Outbreaks were associated with milk sold at local markets and affected both children and elderly people.	[5]
Canada (2018)	350	Children under 5 years	Contaminated salad	Microscopic examination and ELISA*	*C. hominis*	Infected food handlers were traced as sources of contamination.	[9]
Australia (2001)	8	Children	Unpasteurized milk	Microscopic examination and ELISA*	*C. parvum*	Commercial unpasteurized milk was the only exposure with the verified identification of *Cryptosporidium*; the OR* of exposure was 32.7.	[206]
Australia (2020)	300	Children (0–5 years)	Contaminated salad	IFAT	*C. parvum*	Cases were traced to a restaurant with poor hygiene practices.	[207]
Australia (2018)	200	Adults	Contaminated fruit juice	Microscopy	*C. parvum*	Juices were contaminated during processing.	[208]

PCR*, polymerase chain reaction; nPCR*, nested polymerase chain reaction; ELISA*, enzyme-linked immunosorbent assay; RFLP*, restriction fragment length polymorphism; IFAT*, immunofluorescence assay technique; OR*, odds ratio. N/A, Not Available (The exact oocysts count is not available).

Among the 40 recorded cryptosporidiosis incidents in the past 10 years, only 13 are foodborne, while 27 waterborne infections have been documented [10]. This could be because food is far less likely to be contaminated than water, and also, oocyst survival is rare on food due to possible freezing, heating, and desiccation. Tracking foodborne infections is difficult and usually impeded by the possibility that the food item of concern has been eaten and is hence unavailable for investigation, as well as the absence of suitable investigation tools, as some incidents are more prevalent, while others appear sporadic [9]. A comparison between waterborne and foodborne outbreaks reveals an interesting feature; waterborne outbreaks of cryptosporidiosis usually result from *C. hominis*, whereas foodborne outbreaks usually result from *C. parvum* (zoonotic potential). Of the 27 waterborne outbreaks recorded over the past ten years, 6 of them included *C. parvum* as a causative agent, and only 1 case of *C. hominis* was recorded in 13 foodborne outbreaks [10]. Thus, evaluating this hypothesis could represent an innovative research study area for future scientists.

## 9. One Health Approach to Combating Cryptosporidiosis

The literature proves that *Cryptosporidium* oocysts cause foodborne and waterborne diarrheal outbreaks among humans globally and are released into the atmosphere mostly by cattle and most certainly by wild ruminants. The potential of cattle to transmit several species of *Cryptosporidium*, particularly *C. hominis*, emphasizes a major public health hazard associated with possible interactions (Figure 3) [65]. For instance, in Sweden, on a farm, *C. meleagridis* 70 kDa Heat Shock Protein (hsp70) gene sequences were found in samples from humans and chickens, indicating zoonotic potential. An infected calf can produce 30 billion oocysts within a period of 1 to 2 weeks, which are environmentally resilient and may persist for an extended time outside the host, especially in wet surroundings and agricultural farms with infected animals [96]. In addition, zoonotic cryptosporidiosis from sheep has been demonstrated in several epidemics linked with visiting farms with infected animals [209]; also, several cases of waterborne infections of cryptosporidiosis in the UK have been associated with sheep as the reservoir of parasitic infection [210]. Therefore, *Cryptosporidium*, due to its zoonotic transmission, spreads via the environment among animals and humans [31].

Due to its zoonotic transmission, quick action is essential in mitigating this large-scale spread, especially through drinking water and contaminated edible food items. Interactions among all healthcare professionals, such as veterinarians, healthcare personnel, and public health service administrators, can assist in pathogen control by improving the training system, state of consideration, law, and organizational frameworks [211]. The One Health concept was originally suggested to combat cryptosporidiosis as effectively as other infectious diseases [23,211] through interactions among specialists working in different sectors, such as doctors, veterinary surgeons, public health specialists, ecologists, diagnosticians, governments, legislators, pharmaceutical corporations, and epidemiological and social researchers. The One Health strategy consists of (i) raising awareness among the public regarding cryptosporidiosis and its means of spread, (ii) breaking down the transmission routes, (iii) conducting epidemiological studies to estimate the associated risk factors, (iv) the usual monitoring, (v) treating infected animals to reduce incidents in humans, and (vi) educating healthcare professionals and veterinary specialists regarding the handling and diagnosis of the infection.

## 10. Preventive and Control Measures

Due to the lack of effective medications against *Cryptosporidium* spp., precautionary steps need to be considered, but currently, there is no vaccine against *Cryptosporidium*. The preventive measures include washing hands after using the bathroom, handling contaminated food items, and changing diapers; cleaning and disinfecting surfaces contaminated with infected individuals’ feces; boiling water for 3 min at an altitude greater than 6500 feet (exposing oocysts to temperatures greater than 60 °C); and avoiding the usage of untreated water. Regarding food items, raw and unpasteurized food products and fresh food items washed in contaminated water should be avoided. The implementation of legislative measures regarding traveling is vital in safeguarding the health of visitors and the local population. Contaminated drinking water and swimming pools have proven to be a major contributor to the spread of cryptosporidiosis. Adequate oocyst elimination is not achieved in solitary swimming pool flow via filtration systems, so individuals may be exposed to cryptosporidiosis epidemics. To eliminate them all, the water in the swimming pool might have to be cleaned several times. Other control measures like ozone treatment can also be helpful in this regard.

Acidic pH, thermophilic and mesophilic digestion in the absence of oxygen, composting slurry storage, etc., reduce oocysts’ burden [178,212]. One of the present keys to trash disposal is the shift from a linear to a circular economy (CE) approach [213]. The concept of the circular economy should be implemented in the regulation of trash, especially agricultural debris, to allow these sectors to continue their activities in the future [214]. Other preventive measures regarding animals include rotational grazing, the provision of good-quality colostrum to newborns within a few hours after birth, the separation of infected animals from healthy ones for at least one week after relief from diarrhea, steam-cleaning technique, the disinfection of contaminated surfaces using H_2_O_2_, and incorporating vegetative filters strips to avoid cryptosporidiosis [215]. Epidemiological investigations offer considerable value, as they provide helpful evidence on the causes and modes of transmission of cryptosporidiosis in both animals and humans [216]; hence, the implications of the One Health concept are of great significance in preventing and controlling this disease.

## 11. Future Perspectives

Although the immunofluorescence antibody test (IFAT) is a reliable diagnostic technique, as stated in the ISO Standard [217], its high cost and impracticality for routine testing calls for a more feasible identification technique. Shifting to methods such as PCRR-based assay targeting [218] the gp60 gene can enhance routine testing and surveillance [219]. The use of next-generation sequencing (NGS) can lead to improved diagnostic accuracy and the estimation of epidemiological trends [220]. Further studies investigating the role of the gp60 protein in interactions with the host can pave the way to understanding how variations in this glycoprotein affect host vulnerability and pathogenicity in *Cryptosporidium* [221]. *C. bovis* and *C. xiaoi* both seem to have confined host ranges; however, more investigation is integral in estimating the zoonotic risk of these widespread livestock species.

By applying advanced techniques like metagenomics, relative variations in gut microbiota can be studied during *Cryptosporidium* infection, which can help reveal the host defense mechanism against this parasite and, hence, new potential therapeutic targets can be found [221]. Additionally, genetic manipulations using CRISPER/Cas9 technology can be used to determine the role of different genes in the survival of parasites, which could lead to the development of vaccinations and targeted therapies against it [90]. In this regard, microRNA regulation also seems to serve a significant role in host cell defense against the infection of *Cryptosporidium* [222,223,224], and additional investigations in this regard can lead to the development of a vaccine against this protozoan parasite. Due to increasing concerns regarding anthelmintic resistance, exploring the anti-*Cryptosporidium* mechanism of different herbs could provide new therapeutic avenues [225,226]. However, we need more systematic and target-based studies [31]. In addition, considerable research, such as more details on clinical issues and contact exposures, is required. A more complete molecular knowledge of the relationships among sporadic cryptosporidiosis, epidemic settings, and the simultaneous circulation of zoonotic *Cryptosporidium* will facilitate the formulation of a better, precisely adjusted plan for minimizing the transmission of this infection in the future.

## 12. Conclusions

In conclusion, a variety of *Cryptosporidium* species and genotypes, with a broad host range, represent a major threat to food safety, contaminating the food and water industry, and, because of their zoonotic and anthroponotic potential, they cause an ever-increasing burden for both the public health and veterinary sectors. The large number of foodborne and waterborne outbreaks, not only in underdeveloped and developing countries but also in the developed world, highlights the need to control this protozoan parasite. Current therapeutics and control measures are insufficient to cope with *Cryptosporidium*, and after estimating the recent burden of this protozoan parasite, the development of an effective vaccine, improved control strategies, and a potent drug that is effective both in immunocompetent and immunocompromised patients will ensure case reduction and the elimination of outbreaks involving *Cryptosporidium*.

## Figures and Tables

**Table 2:** Cryptosporidiosis in animals, associated contamination sources, and genotypes.

Country (Year)	Animal Infected	No. of Cases	Diagnostic Method	Contamination Source	Prevalence and Primary Symptoms	Genotype and Subtypes of *Cryptosporidium*	Reference
Spain (2018–2022)	Wild boars	498	nPCR* targeting 578 bp sequence of gene encoding SSU* rRNA	Rain-fed crops, forest land	The overall prevalence was 21.7%.	*C. suis*, *C. scrofarum*	[111]
Spain (2004 and 2006)	Lambs and goats	154	DNA extraction, nPCR* of SSU* rRNA gene fragment	Water and feed	Overall prevalence was 32% on lamb farms and 29.5% on goat kid farms.	*C. parvum * IIdA14G1, IIdA15G1, IIdA24G1, IIdA25G1, and IIdA26G1	[112]
Spain (N/A)	Sheep, goats, and cattle	941	PCR to amplify 18S rRNA gene	Drinking water	Oocysts were found in 8.4% of samples from cows, 7.7% of samples from goats, and 5.3% of samples from sheep; the average oocyst count was 5924 oocysts/ gram of feces.	*C. parvum*	[33]
Greece (N/A)	Sheep and goats	530	Microscopic examination and ZN staining* technique	Stagnant water	Overall prevalence was 36.2% in sheep and 65.7% in goats at the farm level.	*C. parvum*	[113]
Greece (2015–2017)	Cattle and sheep	254	PCR* targeting the 18S rDNA and IFAT*	Rivers and irrigation canals	The prevalence rate in calves was 16.7%, and that in lambs was 17.2%.	*C. parvum* *IIaA15G2R1*	[114]
England (2021)	Calves (<6 weeks of age)	28	IFAT*, microscopic examination, and duplex PCR*	Milking machines	Small-scale traditional pasteurization techniques carry more microbial risk than commercial dairy farming.	*C. parvum * IIaA19G1R1	[115]
Algeria (2017 and 2021)	dromedary	63	PCR* and qPCR*	Stool, soil water	Overall prevalence was 7% in camels; none of the animals showed any symptom of diarrhea at the sampling time.	*C. parvum* IIaA15G2R1, IIaA17G2R1 and IIaA18G2R1	[116]
Kuwait (2014–2015)	Sheep and goats	54	ZN staining*, ELISA*, PCR*, and RFLP* analysis of SSU rRNA gene	Sand, feed, and water troughs	Overall, the prevalence was higher by ELISA* than ZN staining* in sheep and goats.	*C. parvum* *IIaA15G2R1* and *IIdA20G1*	[117]
Saudi Arabia (2014–2015)	Sheep, goats, and dromedary	179	ZN staining* and ELISA*	Soil and water	The presence of *Cryptosporidium* oocysts in fecal samples from sheep was 22.2%, from goats was 10.3%, and from camels was 22.4%.	*C. hominis*	[118]
Oman (1998)	Goats (6 months of age)	238	MacConkey agar, sheep blood agar, ZN staining*, and microscopic examination	Water	The major symptom among goats was diarrhea; total reported cases of cryptosporidiosis were 46.7%.	*C. hominis*	[119]
China (2018 and 2019)	Neonatal calves	200	PCR* and sequence analysis of SSU* rRNA gene	Water	The infection rate was 60% higher during the outbreak than after the outbreak (30.4%), and the odds ratio of infection was 11.19.	*C. parvum* IIdA20G1	[120]
Pakistan (2015)	Sheep and goats	300	ZN staining* technique	Tap water and canal water	The overall prevalence was 10.5% in water samples, 28% in canal water, and 8% in tap water.	*C. parvum*	[121]
Australia (2018)	Sheep	35	qPCR*	Drinking water, dam water	*Cryptosporidium* was identified in 10.6% of fecal samples; the total oocyst concentration varied from 518 to 2429 oocysts/g of feces.	*C. xiaoi* and *C. ubiquitum * XIIa	[109]
Brazil (2017)	Calves	31	IFAT* and nPCR*	Water contaminated with fecal material	*C. parvum* was identified in 64% of cases; the results highlighted the significance of water purification and the preservation of sources.	*C*. *parvum * IIaA20G1R1, IIaA17G2R2 and IIaA17G2R1	[122]
Brazil (N/A)	Dogs and cats	188	ELISA* and nPCR*	Sewage system water	Overall, the prevalence in dogs was 8.3% and in cats was 5.5%, and 72.7% of cases presented with diarrhea.	*C. parvum*	[123]

PCR*, polymerase chain reaction; ZN staining*, Ziehl–Neelsen staining; nPCR*, nested polymerase chain reaction; qPCR*, quantitative polymerase chain reaction; IFAT*, immunofluorescence assay technique; ELISA*, enzyme-linked immunosorbent assay; RFLP*, restriction fragment length polymorphism; SSU*, small subunit. N/A, Not Available (The exact oocysts count is not available).

## Data Availability

No new data were created or analyzed in this study because it is a comprehensive review.
